# Capacity versus responsibility: Wealth and historical emissions as determinants of support for climate aid policy

**DOI:** 10.1111/bjso.12899

**Published:** 2025-05-19

**Authors:** Christoph Klebl, Samantha K. Stanley

**Affiliations:** ^1^ School of Psychology The University of Queensland Brisbane Queensland Australia; ^2^ UNSW Institute for Climate Risk & Response University of New South Wales Sydney New South Wales Australia; ^3^ School of Psychology University of New South Wales Sydney New South Wales Australia

**Keywords:** climate change, climate finance, climate justice, climate migration, historical emissions, loss and damage, wealth

## Abstract

Across both wealthy and non‐wealthy nations, research finds public support for wealthy countries taking greater climate action. However, it is unclear whether this is driven by a belief that wealthier nations have greater economic capacity to respond or a greater historic responsibility for causing climate change. We explore this idea in the context of climate aid policies, which direct support to those most affected by climate change. In a correlational study (*N* = 292, United Kingdom), individuals who believe their nation has greater historic responsibility for climate change showed stronger support for their country providing climate aid. Two experiments provide conflicting findings. In Study 2 (*N* = 366, United Kingdom), we experimentally manipulated national wealth and historical emissions using a fictional nation paradigm and found that wealth was the stronger predictor of support for their country providing climate aid. In Study 3 (*N* = 797, South Africa) we manipulated these factors about participants' own nation and found that neither predicted support for climate aid policy, but both predicted greater support for their country implementing climate mitigation policies. Although higher capacity and responsibility increased support for mitigation policies, further efforts are needed to understand their role in shaping support for climate aid.

## INTRODUCTION

Climate change is disproportionately caused by wealthier nations, yet climate effects are disproportionately felt by poorer nations (Oppenheimer et al., 2014; van Houtan et al., 2021). This disparity in who is most responsible and who is suffering the most is one clear way that climate change is an injustice that must be resolved (Sultana, 2022; see Newell et al., 2021; for a discussion of several other dimensions of climate justice). There are growing international discussions about instituting processes to compensate those most affected by climate change (United Nations Climate Change, 2022), and also to support those who may be displaced by climate change (United Nations Human Rights Committee, 2020). However, there is not yet a consensus about how members of the international community should contribute to such measures, such as whether they ought to contribute in proportion to their historic emissions and thus responsibility for causing climate change, or according to their wealth and thus their financial capacity to contribute (e.g. Biermann & Boas, 2008, 2010; Wyett, 2014). Our research is interested in public opinion on these policies. We examine whether public attitudes towards climate aid^1^ policies depend on beliefs about historic emissions and national wealth.

### Climate change as an injustice

High greenhouse gas emissions are causing climate change, which brings a range of devastating effects to people and planet (IPCC, 2023). At present, wealthier nations in Europe, North America, and Oceania have much higher per‐capita carbon dioxide emissions than countries in Africa and South America, where on average emissions are lower than would reflect their ‘fair share’ of global emissions based on the size of each regions' population (Ritchie, 2023). However, there is also a disparity between nations in *historical* emissions. This is because economic growth has generally increased alongside greater consumption of resources and thus emissions (Stern, 2017; Wiedenhofer et al., 2020), meaning that wealthier nations typically have higher cumulative emissions, and hence have had greater contributions to causing climate change than nations lower in wealth. For example, Ritchie's (2019) analysis found that the United States has the world's highest historical emissions, emitting approximately 400 billion tonnes of carbon dioxide alone since 1751. This is twice the second highest contributor, China's 200 billion tonnes. By contrast, the entire continent of Africa has contributed 43 billion tonnes. Despite this disparity in historical emissions, nations and people who have contributed most significantly to climate change will typically escape the worst of its effects (Samson et al., 2011).

The most severe effects of climate change will disproportionately affect those in poorer countries (Oppenheimer et al., 2014), where historical and current per capita emissions are typically low. Suffering is compounded by inadequate resources to adapt to climate change (Taconet et al., 2020). Thus, there is a marked inequality in both the contributors to emissions and consequences of climate change. The Kyoto protocol formally acknowledges one aspect of this disparity by recognizing that ‘developed’ countries have higher historical responsibility for causing climate change, and this idea has been repeated throughout major COP events (Lefstad & Paavola, 2024). Meanwhile, for example, ‘developing’ nations asked at the 1972 Stockholm summit ‘that the nations who caused global environmental problems – and the ones who had the money to address them – should carry the greatest burden in cleaning them up’ (see Klinsky et al., 2017, p. 170). Thus, wealthier nations are sometimes recognized as having a larger role to play in addressing climate injustice owing to their higher responsibility for causing climate change and their greater capacity to fund climate aid measures.

However, not everyone agrees on how to fairly distribute the benefits and burdens from the use and protection of the environment (e.g. for different perspectives on what constitutes justice, see Biermann & Kalfagianni, 2020). For example, wealthier nations have advocated for every country to commit to reducing their emissions, which would place a relatively harsher burden on countries with lower economic capacity (Lefstad & Paavola, 2024). These are just some examples where arguments about nation‐level capacity and responsibility have been leveraged in international negotiations about climate change and well‐publicized in the surrounding media coverage of these events. Whether either (or both) frames are effective in shaping individual beliefs about appropriate policy responses to climate change is an interesting empirical question yet to be examined.

### Addressing climate injustice

Responding to this injustice requires efforts to address the disproportionate burden of climate change on poorer nations (Sultana, 2022). While there are many facets of climate justice approaches, we focus our attention on financial redress and relocation opportunities for those at risk of displacement. One proposed mechanism to compensate those adversely affected by climate change using funds from wealthier nations is the Loss and Damage fund (United Nations Climate Change, 2022). The fund would be available to nations most impacted by climate change and compensate them for losses incurred by climate disasters. A separate fund for adaptation can support nations in strengthening their resilience to climate impacts. However, both funds need greater resourcing. The shortfall in international finance for adaptation measures is estimated to be between five and ten times below the necessary level (United Nations Environment Programme, 2022) and the funds committed to the Loss and Damage fund are well below the estimated level of loss and damage already sustained in developing countries (Wise, 2023).

In some areas, adaptation may not be sufficient to maintain a liveable environment, resulting in displacement. Environmental drivers of migration are difficult to disentangle from other stressors (McAdam, 2012), but displacements linked to environmental conditions are already high, estimated at 23.7 million in 2021 (United Nations Human Rights Committee, 2022). Climate change is expected to increase displacement exponentially. Clement et al. (2021) predict that by 2050, more than 200 million people will be displaced due to climate change, with displacements heavily concentrated in the world's poorest nations. This displacement is typically within countries (i.e. internal migration), though increased international migration is also forecast, such as from low‐lying coastal nations (Storlazzi et al., 2018). Where resettlement has crossed international borders, meta‐analyses indicate that it is mostly to low‐ and middle‐income nations (Hoffmann et al., 2020). International agreements to resettle those displaced internationally do not yet exist.

International climate ‘refugees’ do not meet the criteria for refugee status (Brown, 2008), so existing protections do not apply to them (United Nations, 1951). To summarize the situation: depending on global successes in averting dangerous climate change, there is a growing need to resettle large numbers of people who may become displaced and who are disproportionately concentrated in low‐income nations, and currently being supported by lower‐income nations where border crossings do occur. Again, there are arguments that wealthier nations ought to step up protections of those displaced, such as by Biermann and Boas (2008), who stated: ‘By a large measure, the wealthy industrialized countries have caused most past and present greenhouse gas emissions, and it is thus these countries that have the greatest moral, if not legal, responsibility for the victims of global warming’ (p. 13). Some also argue that financial capacity creates responsibility to act. For example, Wyett (2014) drew on such arguments when suggesting that New Zealand and Australia ought to resettle those displaced within the Pacific.

### Public support for climate aid policy

Understanding public support for climate aid policies is important because public opinion drives policy implementation, particularly in democratic nations (Burstein, 2003; Page & Shapiro, 1983). Little is known about the level of public support for climate aid measures, and whether and how justice concerns contribute to evaluations of policy. The perception of unfairness is an important driver of engagement in collective action movements (Van Zomeren et al., 2008; Walker & Smith, 2002), and perceptions of inequality affect support for climate solutions (Klebl & Jetten, 2024). Seeing those most affected by environmental injustice can also motivate collective action (Lu, 2021), and having a greater awareness of the inequality inherent in climate change predicts greater support for climate aid (Stanley et al., 2025). Therefore, justice concerns are likely relevant to public opinion of climate aid measures.

Previous work has shown that wealthier nations are viewed as having a larger role to play in addressing climate change. Klebl and Jetten (2023) showed that the belief that one's nation is wealthier predicts greater support for structural climate policies and people prefer that wealthier (vs. poorer) nations implement such policies. In two cross‐sectional studies, they found that higher perceptions of ones' own country's wealth related to stronger support for structural climate policies over policies that target individual‐level behaviours. An experiment assigning participants to a wealthy or poor fictional nation identified that people prefer structural climate policies be implemented by wealthier nations than poorer nations. This pattern replicated using quasi‐experimental methods that compared the views of those living in the United Kingdom and South Africa (as a relatively wealthier and less wealthy nation, respectively). As an explanation for these effects, they proposed and found some support for the idea that people view wealthier nations as having a great moral obligation to help solve global issues, and thus support more systemic policy changes. One interpretation is that this perceived moral obligation reflects a recognition of wealthier nations' greater capacity to implement such changes (akin to the ability‐to‐pay rule; Lange et al., 2007). But given that a nation's wealth is associated with its emissions (Stern, 2017), this felt moral obligation could also reflect a recognition of greater responsibility for causing climate change.

To our knowledge, only one study has explored support for climate aid following appeals to nations' capacity or historic responsibility to help. Helbling (2020) conducted a framing study that asked German participants to rate their acceptance of ‘climate refugees’ after random assignment to read one of three arguments in favour of climate migration. Participants either read about the moral obligation to help those in need, or the obligation to help based on nation's capacity (GDP, population size, and past accepted migration numbers), or an argument based on historical emissions. While Helbling found no significant differences in support based on these three different arguments overall, the capacity argument was slightly more favoured, and it was preferred among a non‐environmentalist subsample.

Finally, support for resettling those displaced by climate change, or the provision of climate finance, could also differ between policies that direct support to those most affected within one's own country or overseas. For example, Stanley et al. (2022) showed that US‐based participants had a preference to resettle fellow Americans displaced by climate change than international climate refugees, and this effect replicated with Australian participants (Stanley et al., 2023). Stanley et al. (2025) also found higher support for climate aid policies directing support internally than internationally in both the United States and United Kingdom. It is unclear whether the determinants of support for international versus domestic climate aid differ.

### Current research

While arguments regarding historic responsibility and capacity are already appearing in public discourse and appeals (e.g. Gulliver et al., 2023), there is limited evidence about whether they effectively build support for climate aid policies or potentially backfire, reducing public support. Our research aims to address the question: Is support for climate aid policy dependent on beliefs about a nation's capacity to help or historic responsibility for climate change? We test this question across three studies. In Study 1, we test the extent that beliefs about the United Kingdom's (UK) wealth and contribution to climate change predict support for both domestic and international climate aid policies. In Study 2, we test whether support for climate aid depends on a nations' capacity or responsibility. We use an experimental design in which participants are introduced to a fictitious country, which allows us to systematically vary both national wealth and emissions profile. Finally, in Study 3, we employ an experimental design in South Africa in which we manipulate participants' beliefs about their nation's wealth and emissions to test how these factors affect support for climate aid policies. As exploratory aims, we also examine whether effects of capacity and responsibility beliefs on climate aid support are mediated by feelings of moral obligation to help.

## STUDY 1

In Study 1 (not preregistered), we examined whether beliefs about greater national responsibility for causing climate change and beliefs about greater economic capacity are associated with greater support for international and domestic climate aid. We also tested whether these associations are independent of a general motivation to mitigate climate change and an individual's political orientation.

### Method

#### Participants

We used convenience sampling methods to recruit 300 participants living in the United Kingdom via Prolific, and 296 participants completed the survey. Five participants were removed for failing an attention check; thus, the final sample was 292 (160 women, 130 men, two identified their gender using another term; *M*_age_ = 41.81, *SD* = 12.26, range = 20–66 years). Approval was granted by the Human Research Ethics Committee of the School of Psychology, University of Queensland (2023/HE000388). Participants were given a detailed information sheet and consented to participate in the study. Following the survey, they received a debriefing statement. The data, syntax and materials (for all studies) can be accessed at https://osf.io/smk8z.

#### Measures

To capture beliefs about the United Kingdom's *capacity to help*, we asked participants their opinions on their country's wealth with two items (e.g. ‘To what extent do you believe the UK has more financial resources than other countries?’), and *historic responsibility* with two items (e.g. ‘To what extent do you believe the UK has more responsibility for causing climate change than other countries?’), with agreement rated from 1 (strongly disagree) to 7 (strongly agree).

Support for *international and domestic climate aid* were measured using two items each (e.g. ‘The United Kingdom government resettling people who have had to leave their country due to the effects of climate change’ for international; ‘The UK government providing financial assistance to citizens in the United Kingdom displaced by the effects of climate change (e.g. wildfires, flooding, sea level rise)’ for domestic) rated from 1 (strongly oppose) to 7 (strongly support).

To measure individuals' general motivation to mitigate climate change, we assessed their willingness to make personal *sacrifices for climate change* using a three‐item scale (e.g. ‘I would be willing to accept cuts in my standard of living to limit climate change’), with agreement rated from 1 (not at all) to 7 (very much so). *Political orientation* was measured according to both political beliefs on social issues (e.g. immigration, same‐sex marriage, abortion) and economic issues (e.g. social welfare, government spending, tax cuts), from 1 (left‐wing) to 7 (right‐wing). The study also included questions on unrelated topics for another study, such as philanthropy and carbon offsetting (Klebl et al., 2024; see the https://osf.io/smk8z for a full list).

### Results

Correlations in Table 1 show that beliefs about greater national responsibility for causing climate change are significantly related to greater support for climate aid, both domestically and internationally, and beliefs about greater national capacity relate only to greater support for international climate aid. Support for international and domestic climate aid were positively correlated, indicating that stronger support for one form was associated with stronger support for the other, although mean scores reflected a tendency to more strongly support domestic than international climate aid policy (see Data S1 for paired samples *t*‐tests comparing support for domestic and international climate aid in each study). Greater willingness to make personal sacrifices for climate change was significantly related to greater support for domestic and international climate aid. Political orientation is also relevant, with social and economic conservatism related to lower beliefs of the nation's capacity and responsibility, and lower support for climate aid policy.

**TABLE 1:** Descriptive statistics and correlations among variables in Study 1.

	1	2	3	4	5	6	7
1. Capacity	*α* = .90						
2. Responsibility	.25***	*α* = .77					
3. International climate aid	.27***	.58***	*α* = .86				
4. Domestic climate aid	.11	.35***	.57***	*α* = .74			
5. Social conservatism	−.24***	−.36***	−.56***	−.34***	–		
6. Economic conservatism	−.16**	−.36***	−.45***	−.31***	.79***	–	
7. Climate sacrifice	.25***	.47***	.54***	.36***	−.40***	−.29***	*α* = .92
*M*	4.94	4.50	4.51	5.21	3.24	3.38	3.75
*SD*	1.30	1.41	1.58	1.22	1.45	1.28	1.73

**p < .01; ***p < .001.

Table 2 presents findings from multiple linear regressions. Both viewing the United Kindom as having stronger economic capacity and greater responsibility for causing climate change relate to higher support for international climate aid (explaining 35% of the variance in support). When examined together with our control variables, historic responsibility and willingness to sacrifice for the climate are significant predictors of higher support and economic political conservatism predicts lower support; beliefs about economic capacity no longer significantly predict support for international climate aid. Only responsibility beliefs predicted stronger support for domestic climate aid (explaining 12% of the variance), and this effect remained when adding our control variables, which additionally identified economic conservatism as a predictor of lower support.

**TABLE 2:** Standardized regression coefficients predicting support for climate aid policy (Study 1).

	International climate aid	Domestic climate aid
Step 1	*R* ^2^ = .35***	*R* ^2^ = .12***
Capacity	.13*	.02
Responsibility	.55***	.34***
Step 2	Δ*R* ^2^ = .08	Δ*R* ^2^ = .02
Capacity	.04	−.03
Responsibility	.34***	.18**
Social conservatism	.02	−.08
Economic conservatism	−.34***	−.14
Climate sacrifice	.24***	.21**

*p < .05; **p < .01; ***p < .001.

### Summary

In summary, we found that people who believe that their country has greater historical responsibility for causing climate change are more likely to support both international and domestic climate aid, even when controlling for individuals' willingness to make personal sacrifices for climate change, political orientation, and beliefs about economic capacity. Beliefs about economic capacity, however, were not a significant independent predictor of support for climate aid.

## STUDY 2

We aimed to build on these correlational findings by conducting a preregistered (https://osf.io/bq7er) experiment. In it, participants were immersed in a fictional world and read about disparities in capacity and historical emissions in the six countries of Bimboola. Participants were assigned to the nation of Hima, with random assignment meaning that the description of Hima varied in both wealth (high vs. low) and historical emissions (high vs. low). Thus, while Study 1 participants shared their preexisting ideas about their real nations' wealth and responsibility for causing climate change, Study 2 participants responded about their level of support for climate aid policies as a citizen of Hima, where these details were experimentally varied to induce differences in participants' beliefs about their (fictional) nations' capacity and responsibility.

### Method

#### Participants

We conducted a small pilot test (*n* = 78, see Data S1) to inform a power analysis for a linear regression with three tested predictors (wealth, emissions and their interaction) based on a small effect size (*f* ^2^ = .04), critical alpha of .05, and power of .90. Based on these values, G*Power indicates a minimum sample of 359 is necessary, and we aimed to collect data from 5% above this minimum to account for our preregistered exclusion criteria. We therefore recruited 377 people living in the United Kingdom using convenience sampling via Prolific. A total of 378 participants completed the survey. Following our preregistered exclusion criteria, we removed eight participants who failed the instructional attention check, one who did not consent to the usage of their data, and a further five who failed to correctly identify their country as Hima. Thus, our final sample was 364 (185 women, 178 men, one non‐binary person; age range = 21–81 years, *M* = 47.20, *SD* = 13.50; 92.3% White, 4.4% Asian, 3.0% other ethnicities, 0.03% prefer not to say). The Australian National University Human Research Ethics Committee approved ethical aspects of Studies 2 and 3 and their pilot tests under protocol 2023/322. Prospective participants were presented with a detailed information sheet to help them decide whether to take part in the research. If they consented to take part, they progressed through the online survey and were fully informed via onscreen debrief of the study aims on conclusion of the study.

#### Measures

We adapted Klebl and Jetten's (2023) Bimboola paradigm. In our experiment, prospective participants first learned that they would be introduced to a world consisting of six countries. These countries differed economically based on their GDP and differed in their historical greenhouse gas emissions. They were told that to be eligible to take part in the study, they needed to correctly answer three comprehension questions (see Data S1 for details). Participants then read further information about GDP and historical emissions. They were told they were assigned to the nation of Hima. Participants were randomly assigned to one of four conditions in a 2 (Wealth: high vs. low) × 2 (Historical emissions: high vs. low) between‐subjects factorial design. We presented participants with general information about Hima which was consistent across conditions (e.g. Hima's population size). Participants were then presented with information about the GDP and historical emissions of Hima compared to the other Bimboolean nations, with some key differences in the written descriptions across conditions detailed in Table 3 (for full experimental stimuli, see the OSF page for this project). Participants were also presented with graphs comparing the six nations, with Hima's position shown as either highest or lowest in both GDP and emissions graphs which corresponded to the assigned experimental condition.

**TABLE 3:** Key properties of Hima in each experimental factor.

Factor	Extract from country description
High wealth	**Hima is a very wealthy country**. Despite similar population size, Hima's GDP is 35 times greater than Dinh's GDP. Hima has the **most modern infrastructure**, the **most advanced economy** and the **best employment opportunities** of the six countries
Low wealth	**Hima is a very poor country**. Despite similar population size, Hima's GDP is 35 times smaller than Dinh's GDP. Hima has the **least modern infrastructure**, the **least advanced economy** and the **worst employment opportunities** of the six countries
High emission	For decades, Hima has focused on the **production and sale of fossil fuels**. Hima's production of polluting energy has led to the country having the **world's** **highest historical emissions**
Low emission	For decades, Hima has focused on the **production and sale of renewable energy**. Hima's production of clean energy has led to the country having the **world's lowest historical emissions**

Eight manipulation check questions followed. We asked all participants to rate the extent they agreed that: Hima is a wealthy (poor) country, wealthier (poorer) than other countries in the world, has high (low) historical emissions and higher (lower) historical emissions than other countries in the world, with each rated from 0 (not at all) to 6 (very much so).

Participants then read about environmental problems occurring in Bimboola due to the high greenhouse gas emissions. They were told to imagine they are an elected member of Hima's parliament and rate to what extent they support or oppose Hima implementing a series of policies, from 0 (extremely strongly oppose) to 10 (extremely strongly support). They completed our two main dependent variables: support for *international climate aid* (5 items; *α* = .94; e.g. ‘Contributing to reparations for people worldwide who are most affected by climate change’) and support for *domestic climate aid* (3 items; *α* = .82, e.g. ‘Providing financial assistance to citizens in Hima displaced by the effects of climate change (e.g. bushfire, flooding, sea level rise)’).

Participants learnt that the environmental problems in Bimboola will be severe enough to displace people from their homes, and rated the extent they support or oppose Hima introducing policies to help people displaced by climate change (*resettlement support*; 3 items; *α* = .87; e.g. ‘Offering areas of Hima for people who must leave their own country due to climate change to set up new settlements’) and *requesting aid* from other nations (2 items; *r*(364) = .50; e.g. ‘Asking other countries to resettle citizens of Hima affected by climate change’). They were then asked to what extent they support or oppose Hima introducing policies to further reduce its contribution to climate change (*mitigation policies*; 3 items; *α* = .85; e.g. ‘Increase taxes on Hima industries that have higher emissions’). Next, participants completed three items rating Hima's *moral obligation* (*α* = .85) to contribute to the solution of global issues (e.g. ‘Hima has a moral obligation to do its bit to solve global issues’), from 0 (not at all) to 6 (very much so).

We used the same items from Study 1 to measure political orientation (with clarifying instructions to answer based on participants' real‐life) on both economic and social issues. We included an instructional attention check after this measure, then captured demographics (age, gender, ethnicity, income), and finished with an attention check asking participants to identify the name of their country. After a debrief informing participants of the true study aims, they had the option to withdraw consent for their data to be used in this study. Regardless of their answer to this question, all participants were redirected back to Prolific and compensated for a 10‐min survey at a pay rate of 6GBP/h.

### Results

In total, there were 107 participants in the low‐wealth–low‐emissions condition, 88 participants in the low‐wealth–high‐emissions condition, 64 participants in the high‐wealth–low‐emissions condition and 105 participants in the high‐wealth–high‐emissions condition.^2^

#### Manipulation checks

Participants in high‐wealth conditions believed their country was wealthier and less poor than those in low‐wealth conditions (see Tables 4 and 5). Those in high‐emissions conditions more strongly judged their country to have high historical emissions and less strongly judged it to have low historical emissions, compared to participants in low‐emissions conditions. These results indicate that our experimental manipulations effectively shaped participants' beliefs about the relative wealth and contribution to climate change.

**TABLE 4:** Means and standard deviations of all variables in Studies 2 and 3.

Variables	Study 2								Study 3							
	Low wealth		High wealth		Low emissions		High emissions		Low wealth		High wealth		Low emissions		High emissions	
	*M*	*SD*	*M*	*SD*	*M*	*SD*	*M*	*SD*	*M*	*SD*	*M*	*SD*	*M*	*SD*	*M*	*SD*
Manipulation checks																
Wealthy	0.10	0.59	5.90	0.36	2.28	2.87	3.24	2.93	0.71	1.13	5.13	1.00	2.88	2.41	2.91	2.50
Poor	5.90	0.51	0.06	0.28	3.70	2.85	2.73	2.96								
High emissions	2.74	2.94	3.70	2.85	0.16	0.74	5.87	0.64	2.99	2.76	3.27	2.55	0.71	1.22	5.54	1.01
Low emissions	3.23	2.94	2.26	2.88	5.80	0.90	0.10	0.60								
Main variables																
Domestic aid	7.38	2.14	7.51	1.87	7.68	1.91	7.23	2.09	7.64	2.11	7.62	1.92	7.63	2.00	7.63	2.04
International aid	3.53	2.69	6.10	2.50	4.32	2.80	5.08	2.95	4.87	2.93	5.36	2.56	4.93	2.78	5.29	2.74
Resettlement support	6.42	2.60	6.65	2.37	6.50	2.40	6.55	2.59	5.78	2.62	5.73	2.82	5.63	2.69	5.88	2.75
Requesting aid	7.05	2.51	4.23	2.44	6.80	2.54	4.80	2.78	6.27	2.55	5.73	2.55	6.30	2.39	5.71	2.70
Mitigation	6.03	2.53	6.78	2.53	5.97	2.60	6.73	2.47	6.34	2.73	6.45	2.64	6.25	2.74	6.54	2.62

**TABLE 5:** Manipulation check analyses of Studies 2 and 3.

	*b*	*t*	df	*p*	95% CI	*R* ^2^
Study 2						
Wealthy	5.80	112.69	362	<.001	[5.70, 5.90]	.97
Poor	−5.84	−133.80	362	<.001	[−5.93, −5.75]	.98
High emissions	5.71	79.26	362	<.001	[5.57, 5.85]	.95
Low emissions	−5.58	−59.30	362	<.001	[−5.77, −5.40]	.91
Study 3						
Wealthy	4.41	58.17	795	<.001	[4.27, 4.56]	.81
High emissions	4.83	60.77	795	<.001	[4.67, 4.99]	.82

*Note*: Emission condition was used as an independent variable in regression analyses with emissions variables as dependent variables. Wealth condition was used as an independent variable in regression analyses with wealth variables as dependent variables.

#### Main analyses

**FIGURE 1:**
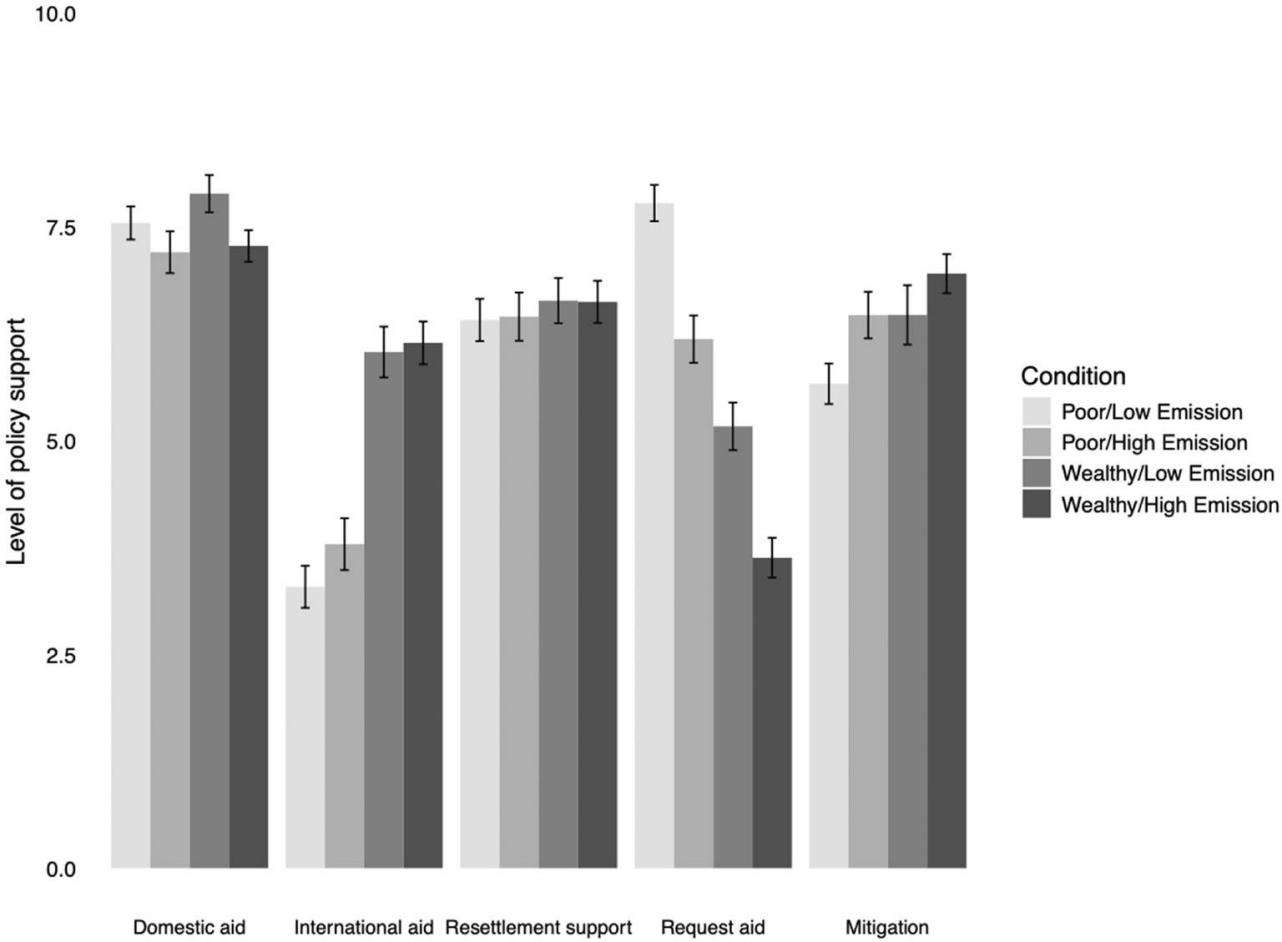
Mean policy support by condition (Study 2).

We conducted linear regression analyses with emission conditions (0 = low emissions, 1 = high emissions), wealth conditions (0 = low wealth, 1 = high wealth) and their interaction as independent variables. Results are presented in Tables 4 and 6, and the mean values of all policy measures by condition are presented in Figure 1. Replicating the pattern in Study 1, mean scores suggest that participants favoured domestic climate aid policy to a greater extent than international climate aid policy (Table 4).

**TABLE 6:** Regression results of Studies 2 and 3.

Dependent variable	Independent variable	Study 2						Study 3					
		*b*	SE	*t*	*p*	95% CI	$\eta_{p}^{2}$	*b*	SE	*t*	*p*	95% CI	$\eta_{p}^{2}$
Domestic aid	Wealth condition	0.34	0.32	1.08	.282	[−0.28, 0.97]	<.01	−0.08	0.20	−0.37	.710	[−0.47, 0.32]	<.01
	Emission condition	−0.37	0.29	−1.29	.197	[−0.94, 0.20]	.01	−0.05	0.20	−0.24	.808	[−0.44, 0.35]	<.01
	Interaction effect	−0.23	0.43	−0.54	.592	[−1.981, 0.62]		0.11	0.29	0.37	.713	[−0.46, 0.67]	
International aid	Wealth condition	2.74	0.41	6.67	<.001	[1.93, 3.55]	.18	0.54	0.28	1.96	.051	[−0.002, 1.08]	.01
	Emission condition	0.51	0.37	1.37	.171	[−0.22, 1.25]	<.01	0.41	0.27	1.50	.134	[−0.13, 0.95]	<.01
	Interaction effect	−0.41	0.56	−0.74	.461	[−1.51, 0.69]		−0.08	0.39	−0.22	.828	[−0.85, 0.68]	
Resettlement support	Wealth condition	0.23	0.40	0.57	.572	[−0.55, 1.00]	<.01	0.22	0.27	0.82	.411	[−0.31, 0.76]	<.01
	Emission condition	0.01	0.36	0.04	.970	[−0.67, 0.72]	<.01	0.51	0.27	1.89	.059	[−0.02, 1.05]	<.01
	Interaction effect	−0.003	0.54	−0.10	.995	[−1.06, 1.05]		−0.54	0.39	−1.39	.165	[−1.29, 0.22]	
Requesting aid	Wealth condition	−2.61	0.37	−7.00	<.001	[−3.34, −1.88]	.19	−0.46	0.25	−1.83	.068	[−0.96, 0.03]	.01
	Emission condition	−1.63	0.34	−4.79	<.001	[−2.29, −0.96]	.07	−0.51	0.25	−2.03	.043	[−1.01, −0.02]	.01
	Interaction effect	0.12	0.51	0.23	.817	[−0.88, 1.11]		−0.17	0.36	−0.47	.641	[−0.87, 0.54]	
Mitigation	Wealth condition	0.80	0.40	2.02	.044	[0.02, 1.59]	.02	0.56	0.27	2.10	.036	[0.04, 1.09]	.01
	Emission condition	0.79	0.36	2.18	.030	[0.08, 1.59]	.02	0.72	0.27	2.70	.007	[0.20, 1.25]	.01
	Interaction effect	−0.30	0.54	−0.56	.558	[−1.36, 0.76]		−0.89	0.38	−2.34	.020	[−1.63, −0.14]	

*Note*: Degrees of freedom (*df*) were 360 for Study 2 analyses. In Study 3, *df* were 792 for international aid analyses and 793 for all other analyses.

##### Support for climate aid

There were no significant effects of emission levels on support for international climate aid, domestic climate aid or resettlement support. Wealth did significantly predict support for international climate aid, where those in the high‐wealth condition expressed greater support for international climate aid than those in the low‐wealth condition (with a large effect size). Wealth did not affect support for domestic climate aid or resettlement support. For these variables, there were no interactions between wealth and historic emissions.

##### Requesting aid

Support for requesting aid was greater in the low‐emission condition than in the high‐emission condition (with a medium effect size). Support for requesting aid was also higher in the low‐wealth condition compared to the high‐wealth condition (with a large effect size). There was no significant interaction effect between the emission conditions and wealth conditions on support for requesting aid.

##### Mitigation policies

Support for mitigation policies was greater in both high‐emission and high‐wealth conditions compared to low‐emission and low‐wealth conditions, respectively (with a small effect size). No significant interaction effect was observed between emission and wealth conditions on mitigation policy support.

##### Mediation analysis

We conducted an exploratory mediation analysis with the wealth condition as the independent variable, support for international climate aid as the dependent variable, and felt moral obligation to contribute to the solution of global issues as the mediator (for full details, see Data S1). There was a significant indirect effect of wealth condition on support for international climate aid through felt moral obligation, *b* = 0.15, *z* = 2.35, *p* = .001, 95% CI [0.021, 0.278].

### Summary

In summary, when assigned to a fictional nation, participants expressed greater support for international climate aid if their nation was described as having high (vs. low) wealth, and this effect was partially mediated by increased feelings of moral obligation to help solve global issues. However, being assigned to a nation with high (vs. low) historical emissions did not affect support for international climate aid, domestic climate aid, or resettlement support. Historical emission and wealth factors both independently predicted support for requesting aid (higher in low emission and low wealth conditions) and support for mitigation policies (higher in high emission and high wealth conditions). Together, our findings suggest that a nation's wealth and emissions profile shape beliefs about the nation's capacity and responsibility to act on climate change, which differently predict policy support depending on whether the policy involves providing aid, requesting aid or reducing emissions.

The effects of responsibility and capacity in shaping climate aid support may also depend on the rating context. The difference in results could reflect that Study 1 examined participants' preexisting views about the UK's responsibility and capacity, whereas Study 2 experimentally varied these factors in a fictional world. We build on these findings in Study 3 by experimentally manipulating beliefs about one's own country's wealth and emissions through comparisons with other nations.

## STUDY 3

In Study 3 (preregistered: https://osf.io/49t5c), we returned to asking participants about policies to be implemented in their own nation. We based Study 3 in South Africa. We selected South Africa because its intermediate position in wealth and historical emissions allowed for meaningful comparisons with both wealthier and less wealthy nations, as well as with countries that have contributed more and less to climate change (Ritchie, 2019; World Bank, 2023). To systematically examine the effects of responsibility and capacity on support for climate aid policies, we used experimentation to manipulate beliefs about South Africa as high (vs. low) in wealth and high (vs. low) in emissions through comparison to other nations. We also included the wider range of climate aid policies from Study 2 to examine unique effects of capacity and responsibility beliefs on support for various forms of aid and climate mitigation. Our hypotheses were the same as in Study 2: we predicted two main effects, where high (vs. low) historical responsibility and high (vs. low) financial capacity would each lead to higher climate aid support, and we were agnostic as to whether these factors would interact.

### Method

#### Participants

We again conducted a pilot test (*n* = 73, described in the Data S1) to inform a power analysis using the same parameters as in Study 2, though aiming to detect a very small effect size (*f* ^2^ = .015). We aimed to recruit a convenience sample of 860 participants via Prolific. A total of 861 participants living in South Africa completed the survey and following our preregistered exclusion criteria, we removed 64 participants. Of these, 56 failed the instructional attention check and eight did not consent to the usage of their data. Thus, our final sample was 797 (389 men, 403 women, 5 non‐binary; age range = 19–73 years, *M* = 29.50, *SD* = 7.77; 80.18% Black, 7.90% Coloured, 7.65% White, 3.51% Indian/Asian, 0.75% not specified).

#### Measures

We designed a variation of the experiment from Study 2 where participants were told about South Africa's economic and environmental performance. Prospective participants first read some information about what GDP measures and what historical greenhouse gas emissions are, and then they were asked for definitions of these constructs as comprehension check questions that were described as necessary to get correct to confirm their eligibility to progress into the study (see Data S1 for details). Participants were then randomly assigned to read further information about South Africa's GDP and historical emissions that involved comparisons to other nations. These comparisons differed depending on random assignment in a 2 (Wealth: high vs. low) × 2 (Historical emissions: high vs. low) between‐subjects factorial design.

In each of the four conditions, the comparison nations were selected based on both GDP per capita data in USD (sourced from World Bank, 2023) that either exceed South Africa's GDP (to make South Africa appear poorer) or were below South Africa's GDP (to make South Africa appear wealthier), and historical emissions data in gigatonnes of CO_2_ (sourced from Ritchie, 2019) that either exceed South Africa's emissions (to make South Africa appear less responsible for causing climate change) or were below South Africa's emissions (to make South Africa appear more responsible). We presented graphs comparing South Africa to these other nations.

Some of the key differences across conditions are highlighted in the text presented in Table 7 (for full experimental stimuli, see the OSF page for this project). South Africa was always described as having a GDP per capita of 133,000 and historical emissions of 16 gigatons of CO_2_ emissions. Our method avoided presenting false information to participants, but it did not allow for perfect experimental control: the distance between South Africa comparator GDP and emission values differed across conditions, and in one condition (high wealth/low emission), we could only identify one comparison country with the necessary GDP/emission profile to allow this comparison. While we endeavoured to use the same countries for GDP and emission comparisons, this sometimes was not possible (though it was in more conditions than it would have been if we used a UK sample for Study 3).

**TABLE 7:** Countries and values used in comparisons to South Africa in Study 3 experimental manipulation.

		Historic emissions (reference: South Africa CO_2_ = 16Gt)	
		High	Low
Wealth (reference: South Africa GDP = 133,000)	High	**Wealth comparisons** Bangladesh (GDP = 5900) Cambodia (GDP = 4400) Madagascar (GDP = 1500) Somalia (GDP = 1100) **Emissions comparisons** Bangladesh (CO_2_ = 1.5Gt) Cambodia (CO_2_ = 0.2Gt) Madagascar (CO_2_ = 0.2Gt) Somalia (CO_2_ = 0.1Gt)	**Wealth comparisons** India (GDP = 6600) Venezuela (GDP = 3600) **Emissions comparisons** India (CO_2_ = 49Gt) Ukraine (CO_2_ = 27Gt)
	Low	**Wealth comparisons** Singapore (GDP = 106,000) Ireland (GDP = 102,500) Portugal (GDP = 33,700) Costa Rica (GDP = 21,200) **Emissions comparisons** Singapore (CO_2_ = 1.8Gt) Ireland (CO_2_ = 2Gt) Portugal (CO_2_ = 2.3Gt) Costa Rica (CO_2_ = 0.3Gt)	**Wealth comparisons** United States (GDP = 63,700) Australia (GDP = 49,800) United Kingdom (GDP = 45,000) South Korea (GDP = 44,200) **Emissions comparisons** United States (CO_2_ = 364Gt) United Kingdom (CO_2_ = 68Gt) Germany (CO_2_ = 126Gt) Japan (CO_2_ = 53Gt)

We then presented the same series of items that followed in Study 2, though with the wording adapted to refer to South Africa rather than Hima. This included two manipulation check items (‘South Africa is a wealthy country’, ‘South Africa is a country with high historical emissions’). Participants read a brief description of environmental problems happening in the world due to the high greenhouse gas emissions and then were asked to what extent they support or oppose South Africa implementing the policies used in Study 2 (international climate aid *α* = .92; domestic climate aid *α* = .76; mitigation *α* = .80; request aid [2 items] *r* = .43, resettlement *α* = .84), and rating their perception of South Africa's moral obligation to help solve global issues (*α* = .87). The same political orientation measure, attention check, and demographic questions tailored to the South Africa context appeared at the end of the survey. Participants were debriefed about the study aims, had the option to withdraw consent, and then were redirected to Prolific and compensated for a 14‐min survey at a pay rate of 6 GBP/h (although the survey content was approximately similar in length as in Study 2, the timing was increased for our South Africa sample based on completion time in our pilot study).

### Results

In total, there were 199 participants in the low‐wealth‐low‐emissions condition, 204 participants in the low‐wealth high‐emissions condition, 199 participants in the high‐wealth‐low‐emissions condition, and 195 participants in the high‐wealth‐high‐emissions condition.

#### Manipulation checks

Manipulation checks confirmed that participants in high‐wealth conditions believed South Africa was wealthier than those in the low‐wealth conditions, and those in high‐emissions conditions judged South Africa to have higher historical emissions than those in the low‐emission conditions (see Tables 4 and 5).

#### Main analyses

**FIGURE 2:**
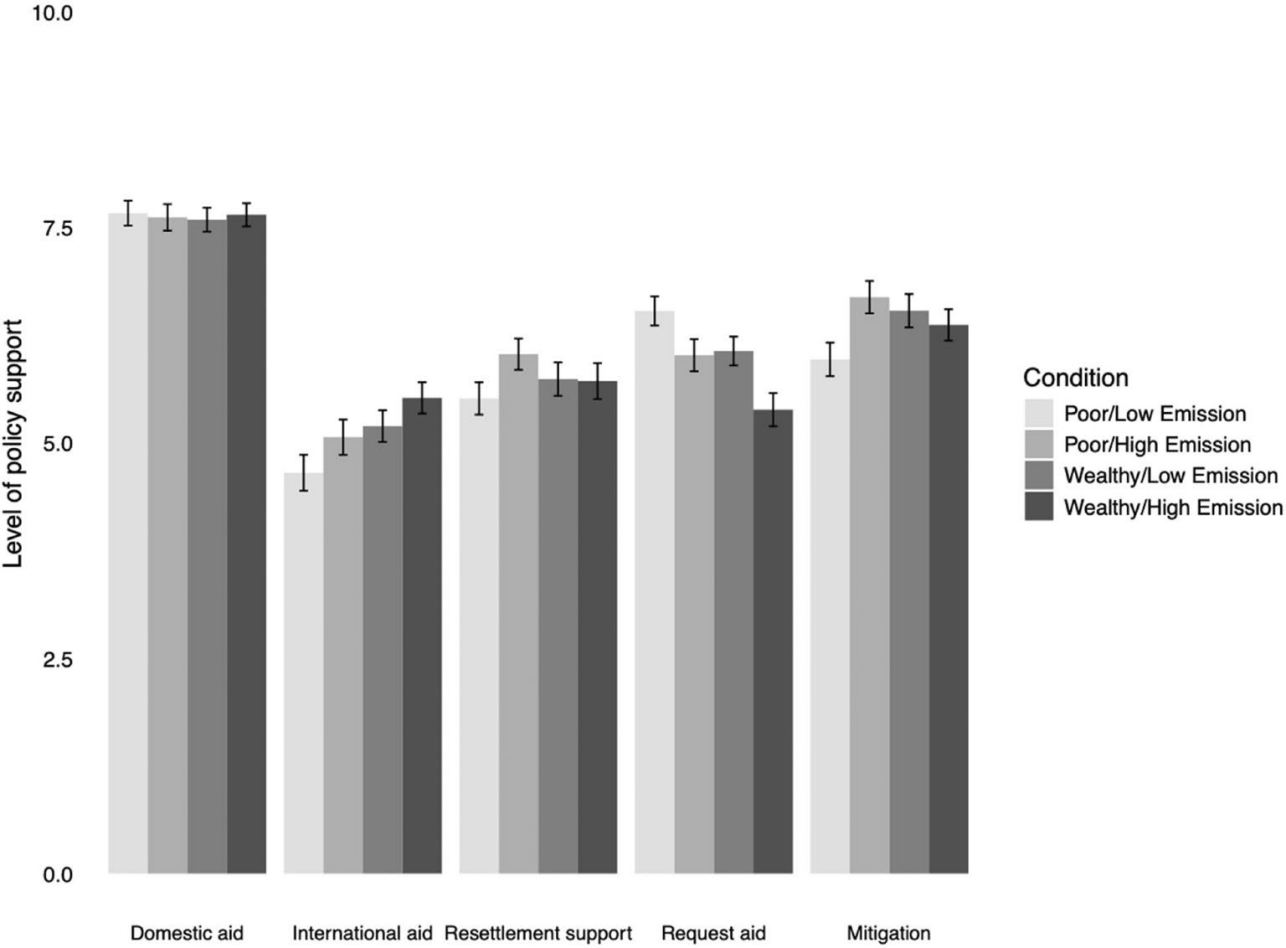
Mean policy support by condition (Study 3). Error bars represent standard errors.

As in Study 2, we conducted linear regression analyses with emission condition (0 = low emissions, 1 = high emissions), wealth condition (0 = low wealth, 1 = high wealth), and their interaction as independent variables. The mean values of all policy measures by condition are presented in Figure 2 and Table 4.

##### Support for climate aid

There were no significant effects of emission levels on support for international climate aid, domestic climate aid, or resettlement support. Wealth had a marginal, but not statistically significant, effect on support for international climate aid (*p* = .051), with a trend suggesting participants in the high‐wealth conditions may show greater support than those in the low‐wealth condition. Wealth did not influence support for domestic climate aid or resettlement support. No interactions between wealth and historic emissions were observed for these variables. Domestic climate aid was more strongly supported than international climate aid.

##### Requesting aid

Support for requesting aid did not significantly differ between the high‐wealth and low‐wealth conditions (*p* = .068). However, there was significantly higher support for requesting aid in the low‐emission conditions than in the high‐emission conditions (with a small effect size). No significant interaction effect was found between emission and wealth conditions on support for requesting aid.

##### Mitigation policies

**FIGURE 3:**
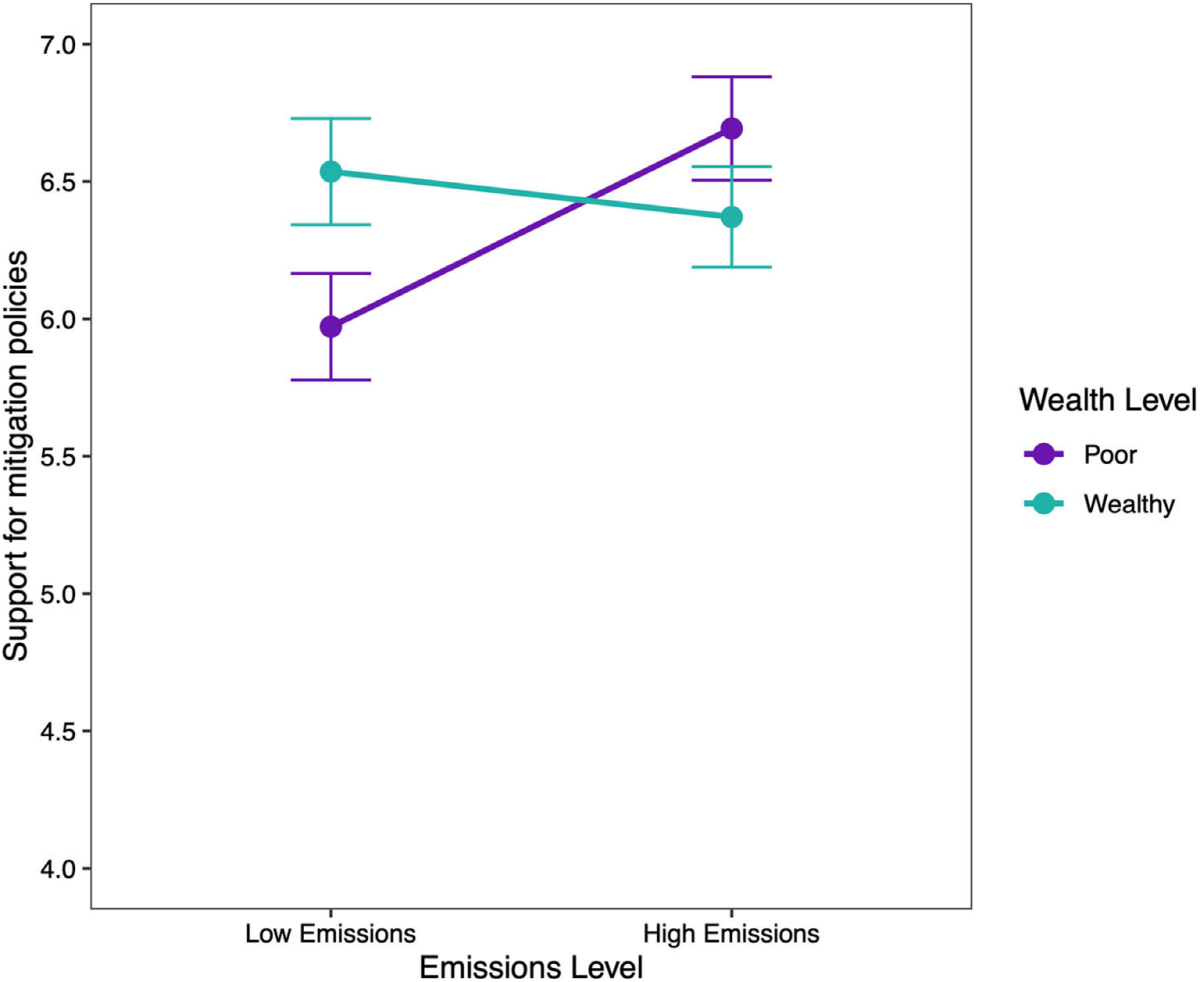
Interaction between emissions level (low vs. high) and wealth level (poor vs. wealthy) on support for mitigation policies. Error bars represent standard errors.

Both emission levels and wealth independently predicted support for mitigation policies (with a small effect size). Participants made to believe South Africa has either high emissions or high wealth expressed significantly greater support for mitigation compared to those made to believe South Africa has low emissions or low wealth. There was also a significant and negative interaction effect between emission and wealth conditions on mitigation policy support (see Figure 3). This interaction suggests that the effect of wealth on mitigation support was stronger when emission levels were low and vice versa.

### Summary

Our experimental manipulation successfully shaped beliefs about South Africa's relative wealth and historical emissions. However, this did not translate into changes in support for climate aid policies—neither presenting South Africa as wealthier nor as historically responsible for emissions affected participants' willingness to support domestic or international climate aid. We did find that when South Africa was presented as having lower historical emissions, participants expressed greater support for requesting aid from other nations, suggesting that people are more inclined to seek international assistance when they view their nation as having contributed less to climate change. Finally, participants were more supportive of climate mitigation policies both when South Africa was presented as having high wealth and when it was presented as having high historical emissions.

## GENERAL DISCUSSION

We sought to understand whether beliefs about national wealth and historical emissions affect support for climate policy responses. We conceptualized these factors as indicators of beliefs about nation‐level capacity to contribute to climate solutions and responsibility for causing climate change, which frequently form the basis of arguments for countries' contributions to addressing climate change and its inequalities (e.g. Biermann & Boas, 2008, 2010; Lefstad & Paavola, 2024; Wyett, 2014). We found mixed evidence across studies for the idea that these factors inform peoples' support for climate aid policy, though consistent evidence that beliefs about higher wealth and higher emissions both predict greater support for climate mitigation policy. This suggests that in responding to climate change, appeals to *both* national capacity (as in Klebl & Jetten, 2023) and historical emissions could build support for emissions reduction. However, more research is needed to understand whether (and when) these factors effectively shape support for climate aid policy.

Alongside critical efforts to reduce emissions is a growing recognition of the need for climate adaptation measures that reduce the negative impacts of climate change. As an area with few insights from psychological research to date, our main interest was in examining beliefs about capacity and responsibility as potential determinants of support for climate aid policies. First, using correlational methods in Study 1, where participants shared their views about their own country's capacity and responsibility, we found that the belief that a nation holds greater responsibility for causing climate change was related to stronger support for both international and domestic climate aid. In Study 2, which was set in a fictional world consisting of six countries that differed in their wealth and historical emissions, participants who were asked to indicate their support as elected members of parliament were more supportive of providing international climate aid when their country had greater (vs. lower) financial resources, while levels of historical emissions did not influence this support. Neither wealth nor emissions influenced support for domestic climate aid. In Study 3, manipulating beliefs about South Africa's wealth and emissions did not significantly affect participants' support for climate aid provision.

These conflicting findings may suggest that beliefs about national capacity and responsibility do not have robust effects on support for climate aid policy, or that these effects are sensitive to the rating context. For example, responsibility was only a significant predictor of climate aid policy when examining pre‐existing views within the United Kingdom, while capacity was relevant only when examining choices to implement international aid policy within a hypothetical world. It is also possible that the rating context moderates these effects. For example, prevailing attributions of blame for causing climate change are perhaps a more proximal construct to support for redistributive action, potentially explaining why these views were more important in Study 1, and receiving information that shaped beliefs about low national wealth could have constrained views about what policy responses are feasible in Study 2.

Another contextual shift was that we moved data collection from the United Kingdom to South Africa in Study 3. Global surveys have shown that people in South Africa are less likely than those in the United Kingdom to prioritize climate change compared to other social and economic issues such as unemployment and poverty (Gebrekal, 2024). Results from our manipulation checks suggested that our experiment successfully influenced beliefs about South Africa's wealth and emissions, however, it is possible that rather than using their beliefs shaped by the information about their nations' capacity and responsibility to inform their support for climate aid policy, South African participants may prefer policy efforts to address issues they perceive to be more pressing. Moreover, given South Africa's GDP per capita is less than half of the global average and has contributed only 1.2% of global historical emissions (Ritchie, 2019; World Bank, 2023), participants' pre‐existing views of their country's wealth and historical emissions may have remained more influential in shaping their policy support, particularly for policies that intersect with issues South Africans consider pressing (Gebrekal, 2024).

Another insight from our studies is that whether capacity and responsibility matter also depends on whether the policy will result in giving aid (as described above) or *receiving* aid. When we measured support for *requesting* aid from other nations in Studies 2 and 3, our findings converged to identify that lower national responsibility beliefs predicted greater support in both studies, with an additional effect of capacity beliefs in Study 2 that did not replicate in Study 3. These findings may indicate that beliefs about lower responsibility for causing climate change increase feelings of entitlement to support from the global community, and in certain contexts, so do beliefs about relative poverty. Applied to international negotiations, this finding may suggest that people feel more comfortable with their nation advocating to receive support (e.g. as beneficiaries of a Loss and Damage fund) on the basis of its low contribution to causing climate change.

Across all studies, we found that participants gave high support for domestic climate aid relative to the scale range. Indeed, support for domestic climate aid was consistently higher than support for international climate aid, suggesting that on average, there is public acceptance of policies that assist fellow citizens affected by climate change, especially as it compares to policies that assist members of the international community affected by climate change. This aligns with previous studies examining public attitudes towards climate migration policy, which identify stronger support for policies that resettle fellow citizens displaced by climate change than members of the international community (Stanley et al., 2022, 2023). The high baseline support also raises methodological considerations, as ceiling effects may have constrained our ability to detect experimental effects on policy attitudes. Future studies could address this by examining more demanding indicators of policy support, such as willingness to take action (e.g. writing a letter to a Member of Parliament advocating for the policy) or acceptance of personal costs to implement these policies. Support for refugee resettlement policies, while above the scale midpoint, was not so high that ceiling effects could plausibly explain null effects. This adds to emerging evidence that determinants of attitudes towards climate migrants may differ from those identified in broader refugee and migration literature (e.g. Stanley et al., 2022, 2023). As climate displacement grows, understanding these unique psychological drivers of support for climate migration policies becomes increasingly important.

Although our main interest was in support for climate aid policy, the addition of mitigation policies in our experimental studies revealed that participants were more supportive of their country (real or fictional) curtailing emissions to mitigate climate change if their nation was framed as having either higher wealth or higher emissions. This finding conceptually replicates and extends Klebl and Jetten's (2023) work, which showed that perceived national wealth increases support for structural climate mitigation policy. In their research, it remained unclear whether people showed greater support for wealthy nations enacting climate policies due to wealthy nations' greater perceived financial capacity to implement climate mitigation policies or due to their perceived historic responsibility for causing climate change. By experimentally manipulating these factors, our work suggests that both beliefs are important determinants of support for climate mitigation policy. The sensitivity of mitigation support to both nations' perceived wealth and emissions may reflect the prominence of these factors in international climate negotiations, where arguments about financial capacity (“ability to pay”) and historical responsibility are central to discussions about national obligations (Ringius et al., 2002). Our experimental manipulations of relative wealth and emissions may have made these established equity principles particularly salient. In contrast, climate aid policies have received comparatively less attention in public discourse (and social science research; Stanley et al., 2022, 2023) and may therefore be less readily connected to these prevailing equity‐based arguments.

Strengths of our study include the use of open science practices, such as open data and pre‐registration of Studies 2 and 3, and the novel focus on beliefs about historical emissions as a potential predictor of support for climate policies to add to the nascent literature on perceived national wealth and inequality (Klebl & Jetten, 2023, 2024). The use of diverse designs to test and conceptually replicate these ideas is a strength, but also a limitation, as it means we cannot definitively explain why findings differed across studies. This presents opportunities for future research to systematically test the possible explanations we put forward above and more directly test how appeals to national capacity versus responsibility influence policy support. Another limitation of our nation‐level analysis is that we did not investigate how these perceptions interact with individual‐level factors. Future research should examine whether beliefs at the nation level are moderated by individual‐level factors. For instance, personal economic circumstances may moderate the relationship between beliefs about national wealth and policy support, particularly for policies that involve resource allocation. Furthermore, while public support drives policy implementation (Burstein, 2003; Page & Shapiro, 1983), it does not always translate into behavioural compliance once policies are implemented (Bickman, 1972). Future research should examine how public opinion influences the adoption of climate aid policies and whether implementation faces particular challenges for policies that require public participation, such as local resettlement programmes. Finally, a limitation of our studies is the use of convenience sampling via Prolific for participant recruitment. While crowdsourcing platforms are widely used in psychological research and provide more diverse participants than traditional university samples, they are not representative of the broader population, including an overrepresentation of White participants in Western samples (Behrend et al., 2011). Future research using representative samples could determine absolute levels of support for climate aid policies in different countries, as well as whether more supportive public attitudes precede or follow the implementation of climate aid policy.

## CONCLUSIONS

Economic growth and emissions generally rise together (Stern, 2017), meaning that emissions are higher in nations with greater income per capita. We examined whether beliefs about one's country's financial capacity and historical responsibility for causing climate change independently shape support for policies aimed at redistributing wealth from higher income, higher emission nations to those most affected by climate change. Our findings present a mixed picture, with several key contributions to answering this question. First, capacity and responsibility beliefs have distinct roles in shaping policy attitudes, each uniquely contributing to support for climate mitigation policy. Second, people were more supportive of requesting aid when they saw their own nation as having lower emissions, suggesting that low historic responsibility may be a more compelling basis for seeking international support. Third, while beliefs about greater historic responsibility predicted stronger support for climate aid, our experimental studies did not establish causal evidence that manipulating beliefs about responsibility or capacity increases support for providing climate aid. Finally, we note that neither highlighting high emissions or high wealth undermined support for climate aid or mitigation policies. Together, these findings suggest the importance of examining how different aspects of nation‐level inequality shape support for various climate policies, and identifying the conditions under which capacity and responsibility framings are most effective.

## AUTHOR CONTRIBUTIONS

**Christoph Klebl:** Conceptualization; methodology; investigation; writing – original draft; writing – review and editing; visualization; formal analysis; project administration; data curation. **Samantha K. Stanley:** Conceptualization; investigation; writing – original draft; writing – review and editing; methodology; project administration.

## FUNDING INFORMATION

Data collection for Study 1 was funded by strategic research funds awarded by the School of Psychology, University of Queensland to Christoph Klebl, and data collection for Studies 2 and 3 was funded by the School of Medicine and Psychology at the Australian National University. Samantha K. Stanley is the recipient of an Australian Research Council Discovery Early Career Award (project number DE240100001) funded by the Australian Government. The views expressed herein are those of the authors and are not necessarily those of the Australian Government or Australian Research Council.

## CONFLICT OF INTEREST STATEMENT

The authors declare no conflicts of interest.

## ETHICS STATEMENT

The ethical aspects of Study 1 were approved by the Human Research Ethics Committee of the School of Psychology, University of Queensland (2023/HE000388) and of Studies 2 and 3 by the Australian National University Human Research Ethics Committee (Protocol 2023/322).

## Supporting information


Data S1:

## Data Availability

The data that support the findings of this study are openly available in OSF at https://osf.io/smk8z.

## Footnotes

^1^
We use ‘climate aid’ as an umbrella term to include approaches that financially compensate those affected by climate losses, fund climate adaptation measures in vulnerable nations and offer resettlement opportunities for those at risk of displacement due to climate change.

^2^
The uneven number of participants across conditions was due to a technical error in the random assignment process. Specifically, we used the “Randomiser” function in Qualtrics to allocate participants to one of four experimental conditions, however we had not ticked the “evenly present elements” option. Thus, the error occurred due to a programming issue. We proceeded with data analysis because regression analyses are generally robust to uneven cell sizes (Kutner et al., 2005). This uneven distribution primarily affects the power to detect interaction effects rather than main effects. Even for the interaction, our power analysis indicated that with our smallest cell (*n* = 64), we maintained adequate power (80%) to detect medium effect sizes of (*f* ^2^ = 0.18).
