# Supplementary material for: Capacity versus responsibility: Wealth and historical emissions as determinants of support for climate aid policy
Source: Br J Soc Psychol. 2025 May 19;64(3):e12899. doi: 10.1111/bjso.12899 (PMC12089692; doi:10.1111/bjso.12899)
Supplement: Supplementary file 1 — Data S1: [file BJSO-64-0-s001.docx]

**Comparing support for domestic versus international climate aid**. We conducted paired sample *t*-tests to examine whether there were differences in the degree to which participants supported domestic climate aid compared to international climate aid. In Study 1, participants expressed significantly greater support for domestic aid (*M* = 5.21, *SD* = 1.22) than for international aid (*M* = 4.51, *SD* = 1.58), *t*(291) = 8.98, *p* < .001. Participants also supported domestic climate aid (*M* = 7.44, *SD* = 2.02) more strongly than international climate aid (*M* = 4.72, *SD* = 2.90) in Study 2, *t*(363) = 18.16, *p* < .001. Finally, in Study 3, participants expressed significantly greater support for domestic aid (*M* = 7.63, *SD* = 2.02) than for international aid (*M* = 5.11, *SD* = 2.77), *t*(795) = 24.39, *p* < .001.

**Pilot Studies**

**Table S1**

|  | Study 2 – pilot study | | | | | | | | Study 3 – pilot study | | | | | | | |
| --- | --- | --- | --- | --- | --- | --- | --- | --- | --- | --- | --- | --- | --- | --- | --- | --- |
|  | Low Wealth | | High Wealth | | Low Emission | | High Emissions | | Low Wealth | | High Wealth | | Low Emissions | | High Emissions | |
| Variable | *M* | *SD* | *M* | *SD* | *M* | *SD* | *M* | *SD* | *M* | *SD* | *M* | *SD* | *M* | *SD* | *M* | *SD* |
| **Manipulation checks** |  |  |  |  |  |  |  |  |  |  |  |  |  |  |  |  |
| Wealthy | 0.00 | 0.00 | 5.87 | 0.34 | 2.97 | 3.02 | 2.91 | 2.96 | 0.79 | 0.92 | 4.54 | 1.20 | 2.41 | 2.11 | 2.58 | 2.23 |
| Poor | 5.97 | 0.16 | 0.10 | 0.31 | 2.97 | 3.02 | 3.09 | 2.96 |  |  |  |  |  |  |  |  |
| High emissions | 3.41 | 2.99 | 3.36 | 2.96 | 0.06 | 0.24 | 5.95 | 0.30 | 2.74 | 2.68 | 3.06 | 2.74 | 0.49 | 0.90 | 5.61 | 0.69 |
| Low emissions | 2.62 | 3.01 | 2.51 | 2.96 | 5.82 | 1.03 | 0.05 | 0.30 |  |  |  |  |  |  |  |  |
| **Main variables** |  |  |  |  |  |  |  |  |  |  |  |  |  |  |  |  |
| Domestic Aid | 7.42 | 2.10 | 7.85 | 1.87 | 7.93 | 1.96 | 7.40 | 2.00 | 7.35 | 2.34 | 7.33 | 1.76 | 7.35 | 2.03 | 7.33 | 2.17 |
| International Aid | 3.33 | 2.57 | 6.87 | 1.84 | 4.86 | 2.92 | 5.28 | 2.80 | 4.62 | 2.65 | 5.37 | 2.34 | 4.67 | 2.67 | 5.29 | 2.34 |
| Resettlement Support | 6.14 | 3.03 | 7.07 | 2.10 | 6.30 | 2.58 | 6.83 | 2.67 | 5.73 | 2.54 | 5.94 | 2.08 | 5.54 | 2.33 | 6.16 | 2.31 |
| Requesting Aid | 6.23 | 2.58 | 4.59 | 2.39 | 5.93 | 2.49 | 5.01 | 2.65 | 5.61 | 2.36 | 6.09 | 2.21 | 5.80 | 2.48 | 5.85 | 2.10 |
| Mitigation | 5.51 | 2.03 | 6.38 | 1.87 | 5.63 | 1.84 | 6.19 | 2.08 | 6.33 | 2.89 | 6.73 | 2.59 | 6.21 | 2.60 | 6.85 | 2.91 |

*Means and standard deviations of all variables in the pilot studies for Studies 2 and 3.*

**Table S2**

*Manipulation check analyses of pilot studies for Studies 2 and 3.*

|  | *b* | *t* | *df* | *p* | 95% CI | *R*^2^ |
| --- | --- | --- | --- | --- | --- | --- |
| **Study 2** |  |  |  |  |  |  |
| Wealthy | 5.87 | 108.30 | 76 | <.001 | [5.76, 5.98] | .99 |
| Poor | -5.87 | -105.80 | 76 | <.001 | [-5.98, -5.76] | .99 |
| High emissions | 5.90 | 93.53 | 76 | <.001 | [5.77, 6.02] | .99 |
| Low emissions | -5.79 | -50.51 | 76 | <.001 | [-6.02, -5.56] | .97 |
| **Study 3** |  |  |  |  |  |  |
| Wealthy | 3.76 | 15.53 | 75 | <.001 | [3.28, 4.24] | .76 |
| High emissions | 5.12 | 27.81 | 75 | <.001 | [4.76, 5.49] | .91 |

**Table S3.** *Regression results of the pilot studies for Studies 2 and 3.*

| Dependent Variable | Independent Variable | Study 2 – pilot study | | | | | | Study 3 – pilot study | | | | | |
| --- | --- | --- | --- | --- | --- | --- | --- | --- | --- | --- | --- | --- | --- |
|  |  | *b* | *SE* | *t* | *p* | 95% CI | *η*_p_² | *b* | *SE* | *t* | *p* | 95% CI | *η*_p_² |
| **Domestic Aid** | Wealth condition | -0.22 | 0.68 | -0.32 | .751 | [-1.57, 1.13] | .03 | -0.91 | 0.65 | -1.41 | .162 | [-2.20, 0.38] | .05 |
|  | Emission condition | -1.10 | 0.64 | -1.72 | .089 | [-2.37, 0.17] | .04 | -0.89 | 0.64 | -1.39 | .168 | [-2.16, 0.38] | .05 |
|  | Interaction effect | 1.14 | 0.90 | 1.26 | .210 | [-0.66, 2.94] |  | 1.93 | 0.95 | 2.03 | .046 | [0.04, 3.81] |  |
| **International Aid** | Wealth condition | 3.00 | 0.77 | 3.91 | < .001 | [1.47, 4.53] | .40 | 0.40 | 0.79 | 0.51 | .612 | [-1.17, 1.98] | .03 |
|  | Emission condition | -0.06 | 0.72 | -0.08 | .939 | [-1.50, 1.38] | .01 | 0.30 | 0.78 | 0.38 | .703 | [-1.25, 1.85] | .02 |
|  | Interaction effect | 0.95 | 1.02 | 0.93 | .358 | [-1.09, 2.98] |  | 0.76 | 1.16 | 0.65 | .516 | [-1.55, 3.06] |  |
| **Resettlement Support** | Wealth condition | 0.29 | 0.90 | 0.33 | .743 | [-1.49, 2.08] | .04 | -0.15 | 0.73 | -0.20 | .839 | [-1.61, 1.31] | .01 |
|  | Emission condition | -0.04 | 0.84 | -0.04 | .967 | [-1.71, 1.64] | .02 | 0.26 | 0.72 | 0.36 | .720 | [-1.18, 1.70] | .03 |
|  | Interaction effect | 1.13 | 1.19 | 0.95 | .346 | [-1.24, 3.50] |  | 0.80 | 1.07 | 0.75 | .457 | [-1.34, 2.95] |  |
| **Requesting Aid** | Wealth condition | -2.44 | 0.84 | -2.91 | .004 | [-4.11, -0.77] | .12 | -0.47 | 0.71 | -0.66 | .511 | [-1.89, 0.95] | .06 |
|  | Emission condition | -1.62 | 0.79 | -2.05 | .043 | [-3.20, -0.05] | .05 | -0.87 | 0.70 | -1.24 | .217 | [-2.27, 0.53] | .05 |
|  | Interaction effect | 1.42 | 1.12 | 1.27 | .209 | [-0.81, 3.65] |  | 2.04 | 1.04 | 1.96 | .054 | [-0.04, 4.12] |  |
| **Mitigation** | Wealth condition | 0.39 | 0.67 | 0.59 | .558 | [-0.94, 1.72] | .06 | -0.13 | 0.86 | -0.15 | .879 | [-1.85, 1.59] | .02 |
|  | Emission condition | 0.14 | 0.63 | 0.23 | .819 | [-1.11, 1.39] | .03 | 0.11 | 0.85 | 0.13 | .897 | [-1.59, 1.81] | .03 |
|  | Interaction effect | 0.84 | 0.89 | 0.94 | .350 | [-0.93, 2.60] |  | 1.19 | 1.27 | 0.94 | .352 | [-1.34, 3.71] |  |

*Note.* Degrees of freedom (*df*) were 74 for the pilot study analyses of Study 2. *Df* were 73 for the pilot study of Study 3.

**Comprehension checks used in Study 2 and 3**

**Study 2:** To progress to the main part of the study, participants were told that they needed to correctly answer three comprehension questions. The first question asked if the study involved six countries. Participants chose between 'True' and 'False', with 'True' being correct. Next, we asked: "The GDP is a measure of:" Participants could pick from 'Economic output', 'Economic inequality', or 'Educational output'. The correct choice was 'Economic output'. The last question was: "Historic greenhouse gas emissions are:" The options were 'Emissions the nation has been responsible for creating throughout history', 'The nation's current emissions', or 'The emissions a nation has removed from the atmosphere throughout history'. The correct answer was that they are the emissions a nation has been responsible for creating throughout history. The survey was configured so that participants were redirected to an end-of-survey message and unable to proceed with the study if they got the number of countries or definition of historical emissions incorrect. However, due to an error in the survey logic, participants were allowed to continue if they selected the incorrect definition of GDP. This error was unlikely to adversely affect our data quality because we had additional checks in place. That is, we removed participants who did not correctly select their country as ‘Hima’ at the end of the survey, and our manipulation checks indicated that our conditions effectively shaped beliefs about Hima’s wealth and historical emissions. Additionally, the pages that followed the comprehension checks included further explanations of GDP to further clarify its meaning.

**Study 3:** We also told participants in Study 3 that they would need to correctly answer two comprehension questions to progress into the survey, which were to correctly select the definition of GDP and historic greenhouse gas emissions. The same error described above persisted in allowing participants to continue with the survey if they selected the incorrect definition of GDP. As with Study 2, the next page of the survey contained further detail on the GDP.

**Table S4**

|  | Study 2 | | | | |  | Study 3 | | | | |
| --- | --- | --- | --- | --- | --- | --- | --- | --- | --- | --- | --- |
| Effects | *b* | *SE* | *z* | *p* | *95% CI* |  | *b* | *SE* | *z* | *p* | *95% CI* |
| IV on M | 0.53 | 0.15 | 3.50 | < .001 | [0.23, 0.83] |  | 0.15 | 0.10 | 1.50 | .133 | [-0.05, 0.35] |
| M on DV | 0.95 | 0.08 | 11.85 | < .001 | [0.79, 1.11] |  | 0.90 | 0.06 | 14.73 | < .001 | [0.78, 1.02] |
| Indirect effect | 0.50 | 0.15 | 3.35 | .001 | [0.21, 0.80] |  | 0.14 | 0.09 | 1.50 | .135 | [-0.04, 0.31] |
| Total effect | 2.58 | 0.27 | 9.43 | < .001 | [2.04, 3.11] |  | 0.49 | 0.20 | 2.53 | .012 | [0.11, 0.88] |
| Direct effect | 2.07 | 0.24 | 8.79 | < .001 | [1.61, 2.53] |  | 0.36 | 0.17 | .039 | .039 | [0.02, 0.70] |

*Mediation analysis results with wealth condition as the independent variable (IV), international climate aid as the dependent variable (DV), and moral obligation as the Mediator (M) for Studies 2 & 3.*

*Note.* CI = 95% Confidence Interval.
